# Case Report: Dual giant neobladder stones weighing 950 grams following 18-year loss to follow-up: surgical management and prevention strategies

**DOI:** 10.3389/fruro.2026.1757555

**Published:** 2026-04-02

**Authors:** Youssef Maachi, Amine Boustani, Amine Salim Lalaoui, Amine Slaoui, Tarik Karmouni, Abdellatif Koutani, Khalid Elkhader

**Affiliations:** Department of Urology, Université Mohammed V de Rabat, Rabat, Morocco

**Keywords:** adequate hydration, aggressive infection control, chronic proteus mirabilis infection, daily bladder irrigation, dual-stone presentations, giant neobladder calculi, giant stones, ileal neobladder reconstruction

## Abstract

**Background:**

Neobladder urolithiasis represents a significant long-term complication following radical cystectomy with orthotopic reconstruction, with incidence ranging from 3-9% for ileal neobladders to as high as 43% for Kock pouch configurations. Giant stones are rare and usually result from prolonged gaps in surveillance combined with inadequate preventive measures.

**Patient presentation:**

This is a case of a 78-year-old male presenting with progressive lower urinary tract symptoms after 18 years without urological follow-up following radical cystoprostatectomy and ileal neobladder reconstruction for muscle-invasive bladder cancer (pT2N0M0) performed in 2006. He had relocated geographically one year post-surgery and stopped all urological care, never performing self-catheterization or bladder irrigation as instructed. Computed tomography revealed two massive neobladder stones measuring 10×8×6 cm and 9×7×8 cm with 1,300 Hounsfield Units density. Open cystolithotomy successfully removed both calculi with a combined weight of 950 grams in 90 minutes with minimal blood loss of 100 mL and no complications. Stone analysis confirmed a pure struvite-carbonate apatite composition secondary to chronic Proteus mirabilis infection. The patient was discharged on postoperative day 3. Follow-up at 1, 3, 6, and 12 months demonstrated complete symptom resolution, stone-free status on imaging, and stable renal function. However, the patient was subsequently lost to follow-up again after 12 months despite multiple attempts at contact.

**Conclusions:**

This case, representing one of the heaviest dual-stone presentations documented in neobladder patients (950g combined weight), demonstrates that complete surgical excision via open cystolithotomy remains the treatment of choice for giant neobladder calculi. The exceptionally prolonged 18-year surveillance gap enabled progressive bilateral stone development, emphasizing the absolute necessity of lifelong structured follow-up programs for all urinary diversion patients. Comprehensive preventive strategies including daily bladder irrigation with 500–1000 mL normal saline, adequate hydration (≥2.5 L daily), aggressive infection control with surveillance cultures every 3–6 months and suppressive antibiotic prophylaxis when indicated, and regular imaging surveillance are essential to prevent such severe complications. Even after prolonged follow-up loss and massive stone development, curative intervention can achieve excellent outcomes when patients subsequently engage with comprehensive preventive protocols, as demonstrated by this patient’s stone-free status during the 12-month compliant period.

## 1 Background

Radical cystectomy with orthotopic neobladder reconstruction represents the preferred surgical treatment for muscle-invasive bladder cancer, offering superior quality of life by preserving body image and maintaining physiological voiding patterns (1, 2). Despite advances in surgical techniques, long-term complications require ongoing surveillance.

The pathophysiology of the formation of neobladder stones may be explained by a triad. One is urinary stasis due to incomplete emptying and presence of residual urine. Second is the presence of chronic bacterial colonization, with the production of urease by bacteria, such as Proteus species, which alkalinizes urine and leads to struvite stones. Finally, the production of excess mucus by the intestinal mucosa serves as a nidus for stone formation. Therefore, a conducive setting is created for the formation of stones, especially in patients who stop the recommended prophylactic interventions such as irrigation and self-catheterization.

Neobladder urolithiasis represents a well-recognized problem with reported incidence varying from 3-9% for standard ileal neobladders to 43% for Kock pouch configurations (3–5). Stone formation results from multiple factors including urinary stasis, metabolic alterations from intestinal reabsorption, chronic bacterial colonization with urease-producing organisms such as Proteus species, excessive mucus production, retained foreign bodies, and chronic inflammation (6).

Most neobladder stones present within the first decade post-surgery, with median detection at 8–11 years (7). However, patients lost to follow-up may develop massive calculi. The largest documented single neobladder stone weighed 5 kg discovered 25 years post-cystectomy (8).

Our case is unique for several reasons: the exceptionally prolonged 18-year surveillance gap represents one of the longest reported; the presence of two separate giant stones with combined weight of 950 grams represents one of the heaviest dual-stone presentations documented; the pure struvite-carbonate apatite composition from chronic Proteus mirabilis infection exemplifies infectious lithogenesis; the association with stable stage 3b chronic kidney disease (eGFR 30 mL/min/1.73m²) demonstrates upper tract complications; and successful management via open cystolithotomy despite advanced patient age (78 years) and renal insufficiency confirms the safety of this approach.

This issue of loss to follow-up has been identified as a global challenge in the management of urinary diversion, and the challenges include socio-economic issues such as relocation, inaccessibility of specialized urological care, financial issues, lack of transfer of care between healthcare facilities, and lack of education for patients about the need for lifelong follow-up. All these challenges affect the vulnerable population and underscore the need for robust recall systems and patient-centered care coordination.

We present this case to emphasize the necessity of lifelong structured surveillance programs, illustrate optimal surgical management techniques, and reinforce evidence-based preventive strategies.

## 2 Case presentation

### 2.1 Patient status

A 78-year-old male patient presented to our emergency urology service in January 2024 with a two-year progressive history of bothersome LUTS, including significantly increased urinary frequency (pollakiuria), painful urination (dysuria), and urgency with occasional urge incontinence. These symptoms were accompanied by chronic lower abdominal pain localized to the suprapubic and hypogastric regions, which had gradually intensified and significantly affected his quality of life, sleep patterns, and the ability to perform routine daily activities.

The patient’s history included muscle-invasive urothelial carcinoma (pT2N0M0), for which he underwent a radical cystoprostatectomy with orthotopic ileal neobladder reconstruction at another institution in 2006. In the postoperative period, he was seen during initial follow-up visits only in the first year and was extensively counseled on lifelong urological surveillance, routine clean intermittent self-catheterization, as well as daily bladder irrigation with normal saline to evacuate the reservoir of accumulated mucus.

In 2007, however, the patient moved to a far geographical region due to professional obligations and then stopped all urological follow-up for almost 18 years. During this long period, he never carried out self-catheterization or bladder irrigation as suggested, because he thought these were not necessary since he could spontaneously void without significant problems.His chronic kidney disease with stable creatinine elevation had been incidentally noted during the preceding two years during routine primary care visits, but no urological evaluation had been pursued.

Family/Social History: No family history of urological malignancies or stone disease. The patient was married, retired from manual labor work, and lived independently. He denied tobacco use and reported minimal alcohol consumption.

### 2.2 Clinical examination

On physical examination, the patient was cooperative, in mild distress due to abdominal discomfort. Vital signs were stable: blood pressure 130/75 mmHg, heart rate 78 beats per minute, temperature 36.8 °C, respiratory rate 16 breaths per minute, and oxygen saturation 98% on room air.

Abdominal examination: The suprapubic distension was visible, with loss of normal contour. The abdomen was soft, without rigidity or guarding. Deep palpation in the hypogastric region revealed tenderness without peritoneal signs. A firm, non-pulsatile mass could be felt in the suprapubic area, from the pubic symphysis to approximately 5 cm below the umbilicus. It was relatively immobile and continuous with pelvic structures.Bowel sounds were normal. The liver and spleen were not palpable.

Digital rectal examination revealed normal rectal tone, no palpable masses, and no blood. The prostate bed (post-cystoprostatectomy) was unremarkable. External genitalia examination was normal. No inguinal lymphadenopathy was detected. Cardiovascular, pulmonary, and neurological examinations were entirely normal.

### 2.3 Clinical data

Laboratory investigations during this period revealed a mild normocytic anemia: hemoglobin was 11 g/dL (reference 13–17 g/dL). White blood cell count was 8,900/mm³, suggesting absence of systemic infection.

Renal function showed chronic kidney disease stage 3b with serum creatinine of 30 mg/L (265 μmol/L, reference <13 mg/L) and eGFR of 30 mL/min/1.73m². These values had remained stable for approximately two years. Serum electrolytes were within normal limits. C-reactive protein was negative.

Urinalysis showed significant pyuria with positive leukocyte esterase (3+). Urine culture revealed Proteus mirabilis at >100–000 colony-forming units/mL sensitive to imipenem, ceftriaxone, and gentamicin.

Transabdominal ultrasound showed marked thickening of the neobladder wall and two large hyperechoic structures producing prominent posterior acoustic shadowing measuring approximately 9–10 cm. Bilateral mild hydronephrosis was observed, more pronounced in the left kidney.

**Figure 1:**
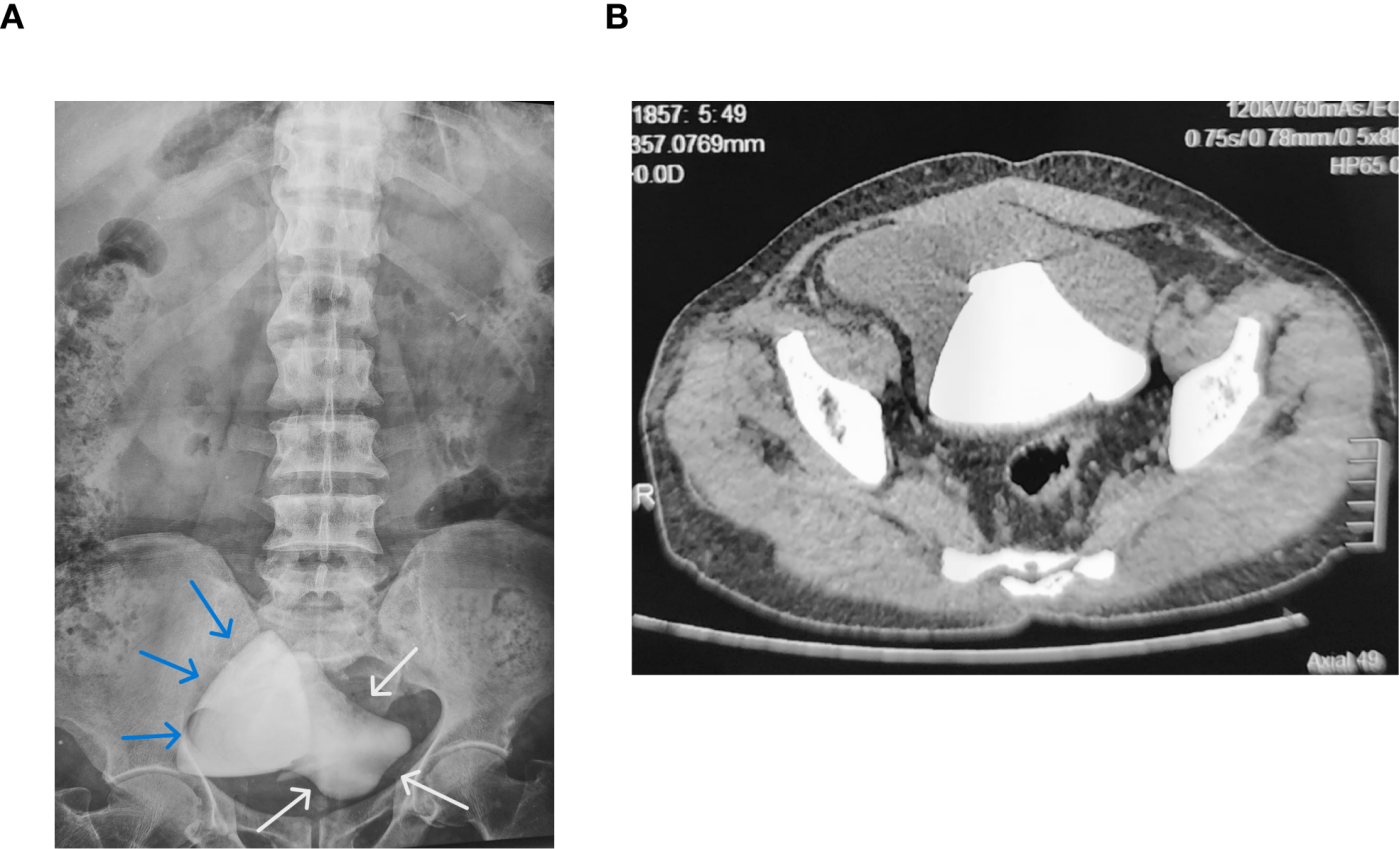
Preoperative imaging of giant neobladder stones. **(A)** Plain abdominal radiograph (KUB view): Two large radio-opaque calcific densities projecting over the neobladder region, consistent with two giant stones. (Blue and White Arrow) **(B)** Axial CT scan: Two high-density stones (1,300 Hounsfield Units) measuring 10×8×6 cm and 9×7×8 cm occupying most of the neobladder capacity.

Plain abdominal radiography confirmed two large radio-opaque densities projecting over the expected neobladder location. (**Figure 1A**).

Computed tomography revealed two high-density stones (1,300 Hounsfield Units) within the neobladder. The first measured approximately 10×8×6 cm, while the second measured 9×7×8 cm. (**Figure 1B**) CT confirmed bilateral mild hydronephrosis, left greater than right, consistent with chronic partial bladder outlet obstruction. No evidence of upper tract stones, neobladder perforation, or fistula formation was observed.

#### 2.3.1 Diagnostic challenges

Initial diagnosis was complicated by the patient’s 18-year absence from urological care, lack of baseline imaging for comparison, and nonspecific symptomatology. The remarkably large stone size initially raised concern for possible malignant mass until imaging clearly demonstrated calcific rather than soft tissue density.

### 2.4 Surgical procedure

The rationale for open cystolithotomy instead of endoscopic management in this case included the following reasons: the enormous size of both stones made endoscopic extraction unfeasible; the presence of bilateral disease necessitated complete clearance; altered anatomy precluded adequate endoscopic access; hard struvite consistency required prolonged lithotripsy time; and, finally, the age of the patient along with associated renal insufficiency favored a single definitive procedure while proven efficacy of open surgery has been demonstrated for giant neobladder stones.

Contemporary minimally invasive approaches, including laparoscopic or robot-assisted cystolithotomy, were also considered but were deemed inappropriate for this patient. This was based on the large stone burden on either side (total stone weight: 950 g), the lengthy duration of surgery required for stone clearance, the small working space within a distended neobladder, and the difficulty of ensuring complete stone clearance via a laparoscopic approach, as well as the patient’s age and renal insufficiency, making a single definitive procedure and minimizing anesthesia exposure advantageous.

Preoperative optimization included intravenous imipenem 500 mg every 6 hours for 48 hours, transfusion of one unit of packed red blood cells (hemoglobin increased from 11 to 12.5 g/dL), and adequate hydration.

The surgical procedure was done under general anesthesia with the patient in the supine position. Then, prophylactic cefazolin 2g was administered intravenously 30 minutes before the incision.A midline lower abdominal incision was made through the previous surgical scar. Careful dissection was performed through moderate intra-abdominal adhesions.

**Figure 2:**
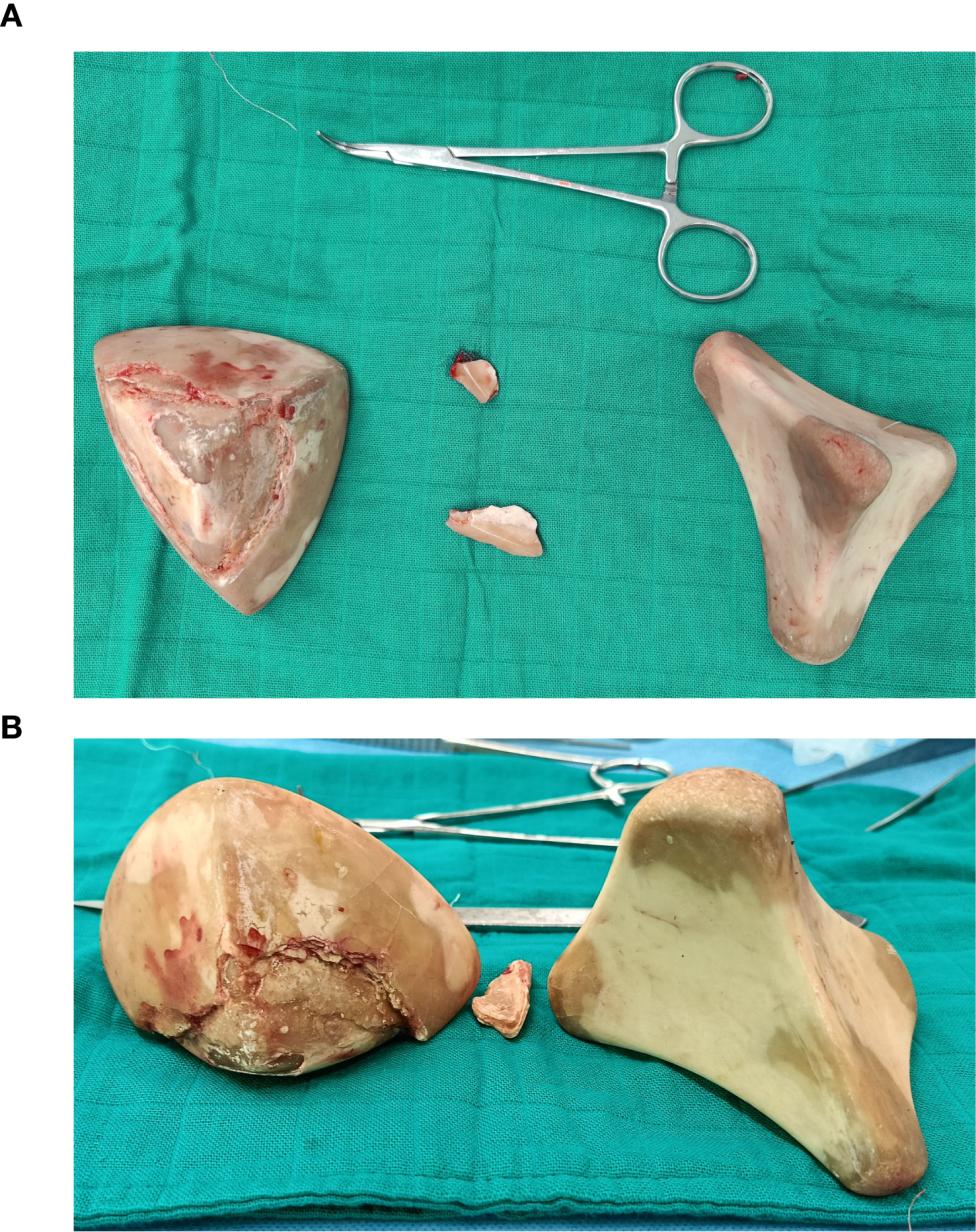
Intraoperative photographs of extracted neobladder stones. **(A, B)** Two massive stones measuring 10×8×6 cm (500g) and 9×7×8 cm (450g) with characteristic brownish-yellow color, hard irregular “staghorn-like” surface, and concentric laminated structure typical of struvite-carbonate apatite composition secondary to chronic urease-producing bacterial infection.

The markedly distended neobladder was identified with thickened but well-vascularized walls. After placing stay sutures, a longitudinal anterior cystotomy approximately 8 cm was performed, revealing the two massive stones. The first stone (10×8×6 cm) weighing 500 grams was carefully extracted. The second stone (9×7×8 cm) weighing 450 grams was similarly extracted intact. (**Figures 2A, B**) Total stone burden was 950 grams. Interior inspection revealed thickened mucosa with chronic inflammation but no tumor recurrence. The ureteroileal anastomoses appeared patent bilaterally.

Copious irrigation with 3 liters of warm normal saline ensured complete fragment removal. The neobladder was repaired using two-layer closure: mucosa with continuous 4–0 Vicryl and seromuscular layer with interrupted 3–0 Vicryl. A Jackson-Pratt drain was placed, and a 22 French three-way urethral Foley catheter was inserted with continuous bladder irrigation at 100 mL per hour.

The abdominal wall was closed in layers. Total operative time was 90 minutes. Estimated blood loss was 100 mL, requiring no intraoperative transfusion. No complications occurred.

Infrared spectrophotometric analysis confirmed composition of struvite (magnesium ammonium phosphate) 85% with carbonate apatite 15% (calcium phosphate), pathognomonic for infectious lithiasis.

### 2.5 Intervention tolerability

The patient tolerated the procedure well and was transferred to the recovery room in stable condition. Pain was well-controlled with intravenous acetaminophen and tramadol as needed, with no requirement for opioid analgesics beyond postoperative day 1. The patient was encouraged to ambulate on postoperative day 1 to prevent thromboembolic complications.

### 2.6 Follow-up

The postoperative course was remarkably uneventful. The pelvic drain output decreased from 50 mL on day 0 to 10 mL on day 2, with drain creatinine confirming absence of urinary leak. The drain was removed on postoperative day 2 because drain output decreased to 10 mL and drain creatinine confirmed absence of urinary leak. Intravenous antibiotics were continued for 5 days postoperatively to ensure adequate tissue penetration, then transitioned to oral ciprofloxacin given stable clinical status and absence of fever.

Oral intake resumed on postoperative day 1. Bowel function returned normally with passage of flatus on day 2. The patient was discharged home on postoperative day 3 with urethral catheter in place, oral ciprofloxacin 500 mg twice daily for 7 days, and detailed instructions.

The catheter was removed on day 10 after plain radiograph confirmed complete stone clearance, ensuring safe resumption of spontaneous voiding.

Comprehensive patient education emphasized: maintaining fluid intake ≥2.5 liters daily; performing daily bladder irrigation with 500 mL normal saline; vigilance for urinary tract infection signs; continuing low-dose nitrofurantoin 50 mg nightly prophylaxis for 6 months; and attending all scheduled follow-up appointments with imaging surveillance every 6 months for the first 2 years, then annually.

Follow-up at 1 month revealed that the patient was asymptomatic, with the resolution of all symptoms. He reported normal voiding patterns. Urine culture was negative for the eradication of Proteus mirabilis infection. His renal function remained stable, with a creatinine level of 28 mg/L and eGFR of 32 mL/min/1.73m².

The patient remained asymptomatic at 3 months. Transabdominal ultrasound did not show evidence of stone recurrence, and there was resolution of bilateral hydronephrosis. Urine culture was still negative.

At the 6-month follow-up there were sustained excellent results, with plain abdominal radiograph confirming continued absence of stones. Renal function remained stable.

At 12-month follow-up, the patient remained completely stone-free both on ultrasound and plain radiography. Renal function was unchanged: creatinine 30 mg/L, eGFR 30 mL/min/1.73m², thus confirming stable chronic kidney disease not progressing.Urine cultures throughout the year were consistently negative. The patient remained highly compliant with all preventive measures.

Unfortunately, he did not show up at the arranged 18-month appointment. Multiple attempts to contact him were unsuccessful. As of manuscript preparation, the patient remains lost to follow-up for the second time.

**Table 1** provides a comprehensive chronological timeline of the 18-year clinical course from initial neobladder reconstruction to stone development, surgical management, and follow-up.

**Table 1:** Clinical timeline.

Year	Event
2006	Radical cystoprostatectomy + ileal neobladder for pT2N0M0 bladder cancer
2007	Geographic relocation → Complete cessation of urological follow-up and preventive measures
2007–2022	Silent bilateral bladder stone formation over 18 years (chronic Proteus mirabilis infection)
2022–2024	Progressive LUTS development (dysuria, frequency, urgency, suprapubic pain)
Jan 2024	Emergency presentation: CT showed 10×8×6 cm and 9×7×8 cm stones, bilateral hydronephrosis
Feb 2024	Open cystolithotomy: 950g stones removed (90 min, 100 mL blood loss, no complications)
Days 2–10	Drain removal (day 2), discharge (day 3), catheter removal (day 10)
Months 1–12	Complete symptom resolution, stone-free imaging, stable CKD, negative cultures
After Month 12	Lost to follow-up again despite multiple contact attempts

Chronological timeline summarizing the 18-year clinical course from radical cystoprostatectomy with ileal neobladder reconstruction (2006) to development of bilateral giant neobladder stones, surgical management in 2024, and postoperative follow-up.

## 3 Patient perspective

This patient described that chronic urinary symptoms and pain have considerably worsened his quality of life, sleep, and daytime performance. Postoperatively, he declared the complete disappearance of symptoms, which he described as “life-changing.” By stopping his follow-up 18 years earlier, he regrets underestimating the silent growth and development of neobladder stones and the value of such simple prophylaxis as daily irrigation. He hopes that his case will raise awareness among other neobladder patients about the importance of lifelong follow-up care and strict adherence to a prophylactic regime.

## 4 Discussion

### 4.1 Neobladder reconstruction techniques and urolithiasis risk

The method of reconstruction of a neobladder also affects urolithiasis risk, and this risk varies depending on different methods of reconstruction. The risk of stone formation is reportedly lower (3 to 9%) for ileal neobladders compared to Kock pouches (up to 43%). This is due to differences in mucus production by intestinal mucosa, metabolic absorptive capacity for electrolytes, geometrical configuration and urinary stasis, and the presence or absence of valves (9, 10). The choice of either ileal or colonic segments also affects urolithiasis risk, and better results are obtained with ileal segments compared to colonic segments. Spherical configurations have a theoretical benefit of reduced surface exposure to urine and decreased pressures compared to cylindrical configurations, potentially reducing stone formation risk (9). In this patient, reconstruction of a neobladder was done in 2006, and this is the best method to prevent urolithiasis. However, complete lack of preventive strategies for 18 years has negated this protective effect. The latest systematic reviews have emphasized that despite advances in surgical techniques, it is essential to adopt all preventive strategies and follow-up, as stone formation risk is present to the extent of 0.5 to 43% depending on the technique and segments of the intestine and preventive strategies (9, 10).

### 4.2 Comparison with previously reported cases

This case presents several features of significant clinical value. The 18-year interval without surveillance allowed progressive bilateral stone development to massive proportions totaling 950 grams. Most giant neobladder stones occur within 9–14 years post-surgery (7–11). Hiruma et al. recently reported the largest single neobladder stone weighing 5 kg at 25 years post-cystoprostatectomy (8). Our case, at 18 years, is unique in its dual-stone presentation with combined substantial weight of 950 grams.

Comparison with previously reported cases (**Table 2**) reveals a progressive spectrum: Hatanaka et al. (12) reported 108g at 9 years, Tiu & Soloway (13) 860g, Nguyen & Choi (14) 770g with 20% struvite composition, and Lu et al. (15) a 9×9 cm stone. Gu et al. (11) described a 903g stone in a 70-year-old female with 4-year follow-up gap, presenting with severe hydronephrosis and renal impairment (eGFR 46 mL/min)—similar to our patient’s stage 3b CKD. These cases demonstrate that prolonged surveillance gaps consistently enable massive stone growth.

**Table 2:** Comparison with previously reported giant neobladder stones.

Author	Year	Age (years)	Sex	Size (cm)	Weight (g)	Time since Surgery	Composition	Management	Follow-up gap	Outcome
Hatanaka et al. (12)	2008	67	M	8 × 7.5	108	9 years	Not reported	Open cystolithotomy	Not reported	Stone-free
Tiu & Soloway (13)	2014	60	NR	12	860	Not reported	Not reported	Open cystolithotomy	Not reported	Stone-free
Nguyen & Choi (14)	2017	64	NR	12 × 9.5 × 7.5	770	Not reported	20% struvite and 80% calcium phosphate	Open cystolithotomy	Not reported	Stone-free
Lu et al. (15)	2022	68	NR	9 × 9	Not reported	Not reported	Not reported	Open cystolithotomy	Not reported	Stone-free
Moazin et al. (17)	2023	25	F	Multiple (14 stones)	Not reported	6 years	Struvite 30%, Carbonate apatite 46%, Whitlockite 24%	Failed endoscopic → Open neocystolithotomy	Not reported	Stone-free
Gu et al. (11)	2023	70	F	13 × 11.5 × 9	903	14 years	Struvite + Calcium phosphate	Open suprapubic cystolithotomy	4 years	Stone-free at 6 months
Hiruma et al. (8)	2025	56	M	21.6 × 15.5 × 18.5	5000	25 years	79% Struvite, 21% Calcium phosphate	Open lithotripsy	Several years	Stone-free at 1 year
Current case	2024	78	M	10 × 8 × 6 and 9 × 7 × 8 (bilateral)	950 (500 + 450)	18 years	Struvite + Carbonate apatite with Proteus mirabilis	Open cystolithotomy	18 years	Stone-free at 12 months

While single large stones predominate, multiple stones occur in 15-20% of cases (16). Moazin et al. (17) reported 14 stones in a 25-year-old female at only 6 years post-surgery, with stone composition (struvite 30%, carbonate apatite 46%) closely mirroring our findings. Their failed endoscopic attempts requiring conversion to open surgery reinforced our decision to proceed directly with open cystolithotomy. In our patient, the similar sizes, identical composition, and absence of foreign bodies suggest synchronous formation around mucus nidi. Complete absence of bladder irrigation over 18 years allowed progressive mucus accumulation as independent nucleation sites.

The pure struvite-carbonate apatite composition with chronic Proteus mirabilis infection is illustrative of infectious lithogenesis. The species Proteus secretes urease, which hydrolyzes urea into ammonia and carbon dioxide; this alkalinizes the urine-usually at a pH above 7.2-and favors supersaturation conditions that allow the precipitation of struvite and carbonate apatite (18, 19). Poor emptying of the bladder-which caused urinary stasis-constant mucus production from the intestinal mucosa, and chronic colonization with Proteus all combined to create the perfect environment for large stone formation over two decades. This case points out that infection control through routine surveillance cultures, prompt treatment of bacteriuria, and consideration of suppressive antibiotic prophylaxis is a key preventive strategy.

This is in association with stage 3b CKD (eGFR 30 mL/min/1.73m²), which raises important considerations regarding upper tract complications. Documented bilateral mild hydronephrosis on CT imaging most likely contributed to obstructive nephropathy due to increased neobladder pressure from incomplete emptying against the massive stone burden. However, two-year stability of renal function suggests chronic factors beyond acute obstruction may be operative, including recurrent subclinical pyelonephritis from ascending infection or chronic vesicoureteral reflux from elevated reservoir pressures. Persistence of stage 3b CKD post-operatively, despite complete stone removal and resolution of hydronephrosis, also suggests irreversible renal parenchymal damage had occurred. This finding emphasizes that prevention and early intervention are paramount, as delayed treatment may allow permanent renal injury even when the obstructing pathology is successfully corrected.

### 4.3 Treatment selection and surgical management

Selection of treatment for neobladder stones is based mainly on stone size, number, location, and composition. Current guidelines recommend endoscopic management as the first-line therapy for stones <4 cm and include transurethral or percutaneous ureteroscopy, pneumatic or laser lithotripsy, and basket or forceps extraction (20, 21). However, the success rates decrease significantly for stones >4 cm, and the anatomical constraints of neobladders, such as altered configuration, multiple bowel folds creating blind spots, absence of normal bladder landmarks, and potential iatrogenic bowel perforation, accentuate the technical difficulties. A number of authors have reported high failure rates and conversion to open surgery when endoscopic management was attempted for giant neobladder stones >10 cm in size (17, 20).

Open cystolithotomy remains the gold standard for giant neobladder stones due to a number of distinct advantages, including: complete visualization of the entire reservoir allowing comprehensive assessment for additional stones, pathology, or complications; ability to extract extremely large or multiple stones intact without prolonged fragmentation; opportunity to repair any incidentally discovered pathology such as fistulas, strictures, or mucosal abnormalities; definitive confirmation of complete stone clearance through direct inspection and irrigation; relatively short operative time compared to prolonged endoscopic procedures, usually in the range of 60–120 minutes; and low rates of retained fragments or recurrence when combined with postoperative preventive measures.

Our case confirms these advantages, with successful complete removal of bilateral massive stones in only 90 minutes, minimal blood loss of 100 mL, no complications, and radiographic confirmation of complete clearance. The patient’s advanced age (78 years) and significant comorbidity (stage 3b CKD) did not preclude safe surgery and support open cystolithotomy as an appropriate option even in higher-risk patients when performed by experienced surgeons.

### 4.4 Prevention strategies and long-term management

Prevention strategies are of utmost importance and should be stressed. Several series have outlined the significant decrease in formation rates seen with routine bladder irrigation following urinary diversion. The seminal publication by Hensle et al. illustrated that with strict reservoir irrigation practices, stone incidence decreased from 42% for non-compliant patients to only 7% for compliant patients (p<0.001); this relative risk reduction was 83% (22).

Based on the available evidence, we would like to propose a comprehensive prevention plan for neobladder patients, including adequate hydration, mucus clearance, infection control, and long-term follow-up. Fluid intake should be at least 2.5–3 L daily, mainly water, in order to keep the urine dilute. Daily bedtime bladder irrigation with 500–1000 mL of normal saline helps remove mucus and debris, and clean intermittent self-catheterization 4–6 times per day is recommended if post-void residuals exceed 100–150 mL. Regular urine cultures every 3–6 months, prompt treatment of bacteriuria—especially due to urease-producing organisms—and low-dose prophylaxis in selected high-risk patients, combined with structured lifelong clinical, laboratory and imaging surveillance, may reduce the risk of stone formation and allow early endoscopic management. Beyond these conventional measures, a holistic patient-centered approach incorporating lifestyle modifications and individualized care strategies may enhance treatment compliance and overall urological health outcomes in neobladder patients (23).

Comparison with published literature provides useful context. Gu et al. reported a 70-year-old female with a single 903g neobladder stone 14 years post-cystectomy, also in a patient lost to follow-up for 4 years (11). Their case featured similar infectious composition and successful open surgical management. Moazin et al. reported a 25-year-old female with 14 separate stones 6 years post-cystectomy requiring conversion from failed endoscopic attempts to open surgery (17).

Our case shares common features with these reports—prolonged follow-up absence enabling massive stone growth, infectious etiology with urease-producing organisms, and excellent outcomes with open surgical management—while adding unique elements including bilateral giant stones with combined 950g weight, exceptionally long 18-year gap without surveillance, and advanced patient age (78 years) with chronic kidney disease. Collectively, these cases uniformly demonstrate that patients lost to follow-up develop the largest stones, that open cystolithotomy achieves superior outcomes for giant calculi, and that comprehensive preventive strategies can successfully prevent recurrence when patients remain engaged with surveillance programs.

### 4.5 Strengths, limitations, and validity

Strengths: This study presents one of the heaviest bilateral neobladder stones (950 g) and the longest period of follow-up (18 years). Objectives of the study have been met by using infrared spectrophotometry to determine the stone composition and the infectious aetiology. Stone-free status has been proven by a comprehensive 12-month follow-up. This study has successfully treated a 78-year-old patient with stage 3b chronic kidney disease.

Validity: This study has been conducted on a single case, and the findings have been validated by using multi-modal diagnostic tools and by comparing the findings with the data available in the literature (**Table 2**).

Limitations: It is not known when the stones started to form during the 18-year period. Since the patient presented as an emergency, metabolic evaluation, including 24-hour urine studies to detect the level of pH, calcium, oxalate, citrate, uric acid, and magnesium, has not been conducted. This would have provided some insight into the synergistic effects of metabolic problems, including hypercalciuria and hypocitraturia. Similarly, urodynamic studies have not been conducted, and this would have provided some objective evidence of the efficiency of bladder emptying and the residual urine volume. Patients have been lost to follow-up after 12 months, and the long-term effects of the preventive measure have not been evaluated.

This study has been conducted on a single patient and cannot be used to formulate any treatment algorithm. Quality of life has not been assessed using any questionnaire.

## 5 Conclusion

This case of bilateral giant neobladder stones totaling 950 grams developing over an 18-year period demonstrates the critical importance of lifelong structured follow-up programs. The successful management via open cystolithotomy with excellent perioperative outcomes confirms this approach as the gold standard for massive neobladder calculi.

Prevention depends on good hydration, daily bladder irrigation, self-catheterization when necessary, rapid treatment of urinary infections—particularly due to urease-producing bacteria—and structured surveillance with recall systems. In practice, this means lifelong organized follow-up, repeated patient education, formal transfer of care in case of relocation, use of open cystolithotomy for giant neobladder stones, and rigorous infection control to prevent struvite stone formation.

Even after long gaps in follow-up and massive stone formation, good outcomes are still possible with appropriate surgery and renewed patient adherence, as shown by this patient’s 12-month stone-free status. This case is both a practical guide for managing giant neobladder stones and a warning about the indispensable need for lifelong surveillance in urinary diversion patients.

## Data Availability

The original contributions presented in the study are included in the article/supplementary material. Further inquiries can be directed to the corresponding author.
